# RETRACTED: Wang et al. Atmospheric Pressure Chemical Ionization Q-Orbitrap Mass Spectrometry Analysis of Gas-Phase High-Energy Dissociation Routes of Triarylamine Derivatives. *Molecules* 2024, *29*, 5807

**DOI:** 10.3390/molecules30224397

**Published:** 2025-11-14

**Authors:** Yi Wang, Shengxiu Wu, Shipan Xu, Xuyang Du, Yuanhui Sun, An Yan, Guijiang Zhou, Xiaolong Yang

**Affiliations:** School of Chemistry, Xi’an Key Laboratory of Sustainable Energy Material Chemistry, Engineering Research Center of Energy Storage Materials and Devices, Ministry of Education, Xi’an Jiaotong University, Xi’an 710049, China

The journal retracts the article titled “Atmospheric Pressure Chemical Ionization Q-Orbitrap Mass Spectrometry Analysis of Gas-Phase High-Energy Dissociation Routes of Triarylamine Derivatives” [1], cited above.

Following publication, the authors requested retraction on the basis of concerns regarding the ownership of the data and contents presented in this article [1]. Xi’an Manareco New Material Co., Ltd. (Xi’an, China) believes that the molecular structures presented in the paper violates their confidentiality agreement with the third party, and the paper needs to be retracted to avoid the risk of infringement.

Adhering to our complaint procedure, an investigation was conducted by the Editorial Office and Editorial Board, which confirmed with Xi’an Manareco New Material Co., Ltd. (Xi’an, China) that the presented data were subject to a confidentiality agreement with a third party and that appropriate permission had not been obtained. Given that this content was deemed to be central to the overall findings presented in this study, the Editorial Board and the Editorial Office decided to retract and remove this article [1], as per MDPI’s retraction policy (https://www.mdpi.com/ethics#_bookmark30). As such, the title, author information, and this retraction note will remain permanently available, and the article itself has been removed. This information has also been transmitted to relevant indexing organizations.

This retraction was approved by the Editor-in-Chief of the *Molecules* journal.

The authors agreed to this retraction.
